# How Does Urban Sprawl Affect Public Health? Evidence from Panel Survey Data in Urbanizing China

**DOI:** 10.3390/ijerph181910181

**Published:** 2021-09-28

**Authors:** Yan Yan, Hui Liu, Canfei He

**Affiliations:** 1 College of Urban Economics and Public Administration, Capital University of Economics and Business, Beijing 100070, China; yanyan@cueb.edu.cn; 2 School of Government, Central University of Finance and Economics, Beijing 100081, China; 3 College of Urban and Environmental Sciences, Peking University, Beijing 100871, China; hecanfei@urban.pku.edu.cn

**Keywords:** urban sprawl, physical health, mental health

## Abstract

This study takes urbanizing China as the research object, employs data from three follow-up surveys conducted by the Harmonized China Health and Retirement Longitudinal Study, and examines the effects of urban sprawl on public health from physical and mental health perspectives. Although urban sprawl does not necessarily increase the risk of each specific type of disease or psychological feeling, it has a significant impact on overall physical and mental health. Further analysis reveals significant heterogeneity in the effects of urban sprawl on the physical and mental health of different groups. Specifically, urban sprawl is detrimental to the physical health of males and females, but only has negative impact on the mental health of females. Younger groups are more vulnerable to physical and mental health damage from urban sprawl relative to middle-aged and older groups. In addition, urban sprawl has a significant negative impact on the health of the low-education group but a very limited impact on the health of the high-education counterpart. From an income perspective, however, the preference for suburban housing among middle- and high-income groups makes their health more vulnerable to the negative effects of urban sprawl than low-income groups living in urban centers.

## 1. Introduction

Urban sprawl has become a common spatial structure worldwide [1,2,3,4]. This phenomenon is characterized by the expansion of urban areas exceeding the growth of the urban population, the dispersal of large numbers of people and economic activities to the suburbs, a decrease in land-use intensity and population density, and a decentralized and polycentric urban form [5,6,7,8]. According to the OECD measurements of 1156 cities in 29 OECD countries, many countries and cities have experienced a dramatic increase in urban sprawl since 1990 [9]. Such low-density urban spatial growth pattern resulted in a range of negative impacts on urban economic, social, and environmental aspects, such as lower productivity and labor wage incomes [10,11], higher energy consumption [12,13], decreased air quality [14,15,16], auto reliance [6,17], and ecosystem fragmentation [18,19]. As a result, urban sprawl has become a hot topic of concern for scholars and policy makers worldwide [1,3,4,20].

Recently, researchers in urban planning and public health have begun to focus on the relationship between urban sprawl and public health, noting that a sprawling urban spatial growth pattern can be harmful to public health [21,22,23,24,25,26,27]. One branch of the literature focuses on the effects of urban sprawl on obesity and finds that people living in sprawl areas are more likely to be obese than those living in compact areas [22,25,28,29,30]. Another stream of literature explores the effects of urban sprawl on mortality and various diseases [25,31] and confirms that residents living in sprawl areas may face higher mortality rates [32,33] and are more likely to develop heart disease [25,34], high blood pressure, and diabetes [25]. By contrast, a compact urban form can reduce cardiovascular mortality [35]. The third branch of the literature examines the impact of urban sprawl on the health care costs of the residents and establishes that urban sprawl increases residents’ health care expenditures [36,37]. Accordingly, the World Health Organization (WHO) advocates the use of urban planning as a tool for high public health [38].

Several studies subsequently explore the mechanisms by which urban sprawl affects public health and verify that urban sprawl can affect public health through reduced physical activity [25,36,39], decreased air quality [14,15,16], pedestrian-unfriendly built environment [1,40,41], pedestrian injuries and fatalities [22,42], and other ways. Urban sprawl can reduce the physical activity of residents, and the lack of physical activity is considered the fourth leading risk factor for death globally and causes 3.2 million deaths annually. Urban sprawl also tends to fragment intra-urban spaces and increases commuting distances for residents, with this occurrence leading to reliance on cars and a reduction in active transport (e.g., such as walking and cycling), which is considered a convenient way to increase physical activity [6,43,44,45]. Furthermore, urban sprawl tends to create built environments that are not friendly for walking, and this situation can lower the probability of resident engagement in physical activity [1,40]. In addition, urban sprawl can lead to a decrease in air quality, a condition which affects the health of residents [14,15,16]. Urban sprawl contributes to increased vehicle emissions [12,13] and rising construction dust [46], both of which can further harm the residents’ health by causing respiratory diseases, cardiovascular diseases, and liver fibrosis [47,48].

Relatively rich research on urban sprawl and public health is available, but some shortcomings and deficiencies are evident in the literature. First, scholars have mostly studied the effect of urban sprawl on public health from the perspective of physical health. Some discussions regarding mental health have emerged in recent years, but they are still very limited. In fact, urban sprawl also affects mental health directly or indirectly. Air pollution caused by urban sprawl can increase oxidative stress and systemic inflammatory responses in humans, directly contributing to depression and cognitive dysfunction and even causing brain damage and dementia [49,50]. Changes in the built environment because of urban sprawl can also increase the risk of depression among residents [51]. Second, existing studies on the relationship between urban sprawl and public health mainly focused on developed countries such as European nations and the U.S. [27,28], with relatively few studies targeting developing countries. China is a typical case of a developing country for studying this issue. As a developing country undergoing rapid urbanization, China’s traditional high-density model of urban spatial development has been disappearing gradually. On the contrary, urban sprawl has become a common phenomenon in its current urban development [4,52]. Although urban sprawl in Chinese cities has been promoted jointly by local states and market forces and simultaneously by real estate developer and industrial manufacturers, both of which are different from western cities, the spatial pattern and its multidimensional impacts are quite similar to those in other countries [53,54]. At the same time, public health has become an important challenge for developing countries. This is the case for China as well. The WHO (2016) reported that mental illness, diabetes, and cardiovascular disease will cause the most economic damage in urbanizing China in the future among all diseases, and this trend is exacerbated by urban sprawl and the lifestyle and the work changes it generates. Third, given the limitation of survey data, most previous research on the relationship between urban sprawl and public health have used cross-sectional data. Nevertheless, cross-sectional data are difficult to control for all unobservable individual differences and are prone to problems such as omitted variables.

Accordingly, this study uses the authoritative data published by the China Health and Retirement Longitudinal Study (CHARLS) to examine the effects of urban sprawl on public health in urbanizing China. The main contributions of this work are as follows. First, this article assesses the effect of urban sprawl on public health from the perspectives of physical and mental health, which are comprehensive viewpoints. Second, this study uses China as a case and thus makes up for the relative lack of such research in developing countries. Third, the data used in this work are tracking survey data, cover three periods (2011, 2013, and 2015), and are comparable between periods. The tracking survey data provide information on the dynamic behavior of individuals and can significantly improve the precision of the estimation. Fourth, this work further explored the heterogeneous characteristics of the effects of urban sprawl on the physical and mental health of different groups in four dimensions: gender, age, education, and income level.

## 2. Research Methods and Data Sources

### 2.1. Model

To test the impact of urban sprawl on public health, this study constructs the following model.

(1) $$PH_{ict}=\beta_{0}+\beta_{1}US_{ct}+\gamma_{1}X_{it}+\tau_{c}+\omega_{t}+\varepsilon_{ct}$$

(2) $$MH_{ict}=\beta_{0}+\beta_{1}US_{ct}+\gamma_{1}X_{it}+\tau_{c}+\omega_{t}+\varepsilon_{ct}$$

where the subscript *i* represents different individuals, *c* denotes city, and *t* denotes waves of survey. $\tau_{c}$ and $\omega_{t}$ refer to the city fixed effects and time fixed effects, respectively. $PH_{ict}$ and $MH_{ict}$ represent the physical and mental health status of respondent *i* living in city *c* for the *t*th waves of survey. $US_{ct}$ represents the degree of urban sprawl in city *c* in year *t*. Referring to the previous literature, the control variables mainly include the following three categories. The first category controls individual demographic variables, such as age, gender, marital status, education level, and employment status. The second category controls lifestyle and health behavior variables, including smoking and alcohol consumption. The third category controls family structure variables, including the number of people living in household, whether any child is co-residing with the respondent, and annual household income. As CHARLS involves longitudinal data, this study refers to Lim and Hong [55] and Wang et al. [56] and employs the generalized estimating equations (GEE) method to examine the relationship between urban sprawl and public health.

### 2.2. Data Sources

The individual-level microdata of middle-aged and older adults used in this paper were obtained from the Harmonized CHARLS database which was provided by the Center for Socioeconomic Research (CESR) at the University of Southern California and has been widely used to study health problems of Chinese residents [57,58]. The raw data for Harmonized CHARLS came from the CHARLS data organized by the National Development Institute of Peking University. CHARLS started the baseline survey in 2011 and conducted follow-up interviews in 2013, 2015, and 2018. CHARLS adopted a multi-stage cluster and stratified probability proportionate to size (PPS) sampling method to conduct the survey to ensure the representativeness and unbiasedness of samples. CHARLS randomly selected residents aged 45 years or older and their spouses for the survey. For ease of use and international comparison, CESR linked the CHARLS data to the variables from the Health and Retirement Study data produced by the RAND Corporation (RAND HRS) and named the outcome as Harmonized CHARLS. The Harmonized CHARLS database combines the CHARLS 2011, CHARLS 2013, and CHARLS 2015 samples, making each wave of survey comparable for easy establishment of panel models. In addition, the Harmonized CHARLS integrates numerous variables on the basis of CHARLS data, with relatively few missing variables and high data quality.

The urban sprawl index was calculated according to the MODIS Global Urban Extent Product (MGUP) data [59] and WorldPop global population density data. To obtain a reliable urban sprawl index, the extent of urban built-up areas and their respective populations needed to be identified. However, the area provided by China’s urban economic statistics may vary in standard from time to time and from city to city. Furthermore, China does not have resident population statistics accurate to the neighborhood scale as the United States does. Therefore, a more precise method is required to measure urban built-up areas and their population. In this research, we use MGUP data to identify the largest patches within the administrative boundaries of each city as urban built-up areas, combine the WorldPop data to calculate the population of each built-up area, and finally compute the urban sprawl index accordingly. MGUP data are widely used in the study of global urban areas [59,60]. MGUP has been validated to be more than 90% accurate in identifying urban boundaries in multiple scenarios [61]. As a class of data reflecting the spatial distribution of population, WorldPop data have higher estimation accuracy and longer duration compared to other gridded population spatial distribution data and is ideal for measuring the spatial distribution of urban populations [62].

We linked individual-level survey data for each city with city-level urban sprawl index for further analysis. This study only retains samples that are 45 years old or older and do not have missing dependent and important independent variables. As urban sprawl mainly affects the health of residents living in urban areas, this study removed the sample of respondents living in rural areas. It is worth noting that although the lifestyle and mobility of residents living in the suburb are affected more severely by urban sprawl than those living in the city center, the latter cannot completely escape from the impacts of urban sprawl because their commuting and everyday life are citywide behaviors which cannot be restrained only in the city center.

### 2.3. Variable Definition

This study used the total number of diseases from which the respondent suffered as a measure of physical health, which is widely used in the previous literature [63,64]. CHARLS asks respondents whether or not a doctor has told them they had a specific disease. The specific diseases include cancer, high blood pressure, diabetes, dyslipidemia, heart problem, stroke, asthma, lung disease, liver disease, kidney disease, stomach/digestive disease, and arthritis. A code of 0 indicates that the respondent does not report having been told by a doctor they have the condition. A code of 1 indicates that the respondent reports having been told by a doctor they have a condition. We summed the respondents’ answers to 12 conditions as a measure of physical health. The final value of $PH_{ict}$ ranges from 0 to 12, with larger values representing poorer physical health.

Previous studies have mostly evaluated the mental health in terms of cognitive ability and depression self-assessment [49,50,51]. Relatively, self-assessment of depression is a more common indicator to measure residents’ mental health. Fortunately, CHARLS offers the short form of the Center for Epidemiological Studies Depression scale (CESD-10), which is widely used in mental health research [57,65,66]. Lei et al. examined the reliability and validity of the CESD-10 scale using CHARLS data and confirmed the validity of the scale in the Chinese population studies [57]. The CESD-10 scale contains three depressive mood items, five somatic symptom items, and two positive mood items. The CESD-10 scale contains three depressive mood items, five somatic symptom items and two positive mood items. CHARLS reports the frequency of the respondents’ feeling over the week prior to the interview, specifically including whether the respondent was feeling depressed, whether the respondent was feeling that everything was an effort, whether the respondent’s sleep was restless, whether the respondent felt happy, whether the respondent felt lonely, whether the respondent bothered by thing that did not usually bother them, whether the respondent felt they could not get going, whether the respondent has trouble keeping their mind on what they are doing, whether the respondent felt hopeful about the future, and whether the respondent felt fearful. When respondents answered to negative mood as “rarely or never (less than once a day),” “some or a little of the time (1–2 days),” “occasionally or a moderate amount of time (3–4 days),” and “most or all of the time (5–7 days),” they were scored 0–3 points. The positive emotions were reverse coded. Referring to the existing literature, this study used the CESD-10 scale to measure the degree of mental health. The score ranges from zero to 30, with higher values representing worse mental health.

Given data limitations, previous literature typically used population density as an approximate measure of urban sprawl. This study adopts the approach from Fulton et al. [67] to reflect the degree of urban sprawl by calculating the ratio of the urban built-up area growth rate and urban population growth rate using the following method. Note that the larger the $US_{ct}$, the higher the degree of urban sprawl.

(3) $$US_{ct}=\frac{A_{ct}-A_{ct-1}}{A_{ct-1}}/\frac{\left(P_{ct}-P_{ct-1}\right)}{P_{ct-1}}$$

where $A_{ct}$ refers to the built-up area at time *t*, and $P_{ct}$ refer to the population within the study area at time *t*.

The control variables are defined as follows. Age indicates the respondent’s age. Gender indicates the respondent’s gender which set to 1 for male and 0 for female. Marriage indicates the respondent’s reported marital status. Marriage is set to 1 for married and 0 for separated, divorced, widowed, and never married. Education indicates the highest level of education the respondent has attained. Education is defined 1 for upper secondary and vocational training and 0 for less than lower secondary education. Education_2 is defined 1 for tertiary education and 0 for less than lower secondary education. Employment refers to the working status. Employment is set to 1 for currently working and 0 for unemployed, retired, or never worked. Smoke indicates whether the respondent reports ever smoking. Smoke is set to 1 for ever smoking and 0 for never having smoked. Drink indicates whether the respondent has had any alcoholic beverages in the past. Drink is set to 1 for having had an alcoholic drink in the past and 0 for never having an alcoholic drink in the past. Hhincome (for household income) indicates the sum of all income at the household level. To make the data comparable, this work uniformly converts annual household income to 2015 prices according to the consumer price index, excluding the effect of price factors. To eliminate the effect of heteroskedasticity, annual household income is taken in logarithmic form in the regression. Hhcoresd indicates whether any child is co-residing with the respondent. Hhcoresd is set to 1 for any child co-resides with respondent and 0 for no child co-resides with respondent. Hhnum indicates the number of people living in household. Table 1 shows the descriptive statistics of the main variables.

## 3. Empirical Analysis

### 3.1. Benchmark Regression Results

Table 2 shows the results of the benchmark regression of the effect of urban sprawl on public health. Urban sprawl has a significant negative effect on the physical and mental health of respondents. As the level of urban sprawl increases, the total number of specific diseases and the level of psychological depression of the respondents rose significantly. Specifically, each 1-unit increase in the urban sprawl index was associated with a 1.1% rise in the number of specific diseases and a 1.2% rise in the CESD-10 scale score which reflects the degree of depression.

The regression results of other control variables are further observed. As age increases, the physical health of the respondents worsens and the likelihood of getting various diseases increases. However, the effect of age on the mental health is not significant, thereby indicating that the mental health of the respondents has little relationship with their age. Compared to females, males have better physical and mental health, a lower risk of developing diseases, and relatively lower degree of depression. Compared to unmarried, widowed, and divorced respondents, married counterparts had better physical health but relatively poorer mental health. Marriage has a protective effect on health and married individuals have better physical health status [68]. Compared to those with less than lower secondary education, respondents with higher levels of education have relatively better mental health but no significant differences in physical health. Studies confirmed that individuals with higher levels of education are more capable of adjusting their behavior and regulating their psychology amid stressful life events and have higher levels of mental health [69]. Compared to people who are unemployed, retired or have never worked, respondents currently working have better mental health. Respondents with drinking and smoking habits are more likely to have a disease, and smoking also has a significant effect on mental health. The negative health effects of drinking and smoking have been confirmed by numerous studies [70,71,72]. Respondents with higher household income had relatively better physical and mental health. Higher income groups generally have a relatively higher quality of life and are more likely to access advanced medical resources and healthy nutrition. Moreover, the accumulation of wealth can affect cognitive functioning and mental health, so higher income groups are physically and mentally healthier [73,74]. Whether or not living with children has no significant effect on respondents’ physical and mental health. With increasing family size, respondents’ physical health rises, but their mental health does not change significantly.

### 3.2. Robustness Test

**Figure 1:**
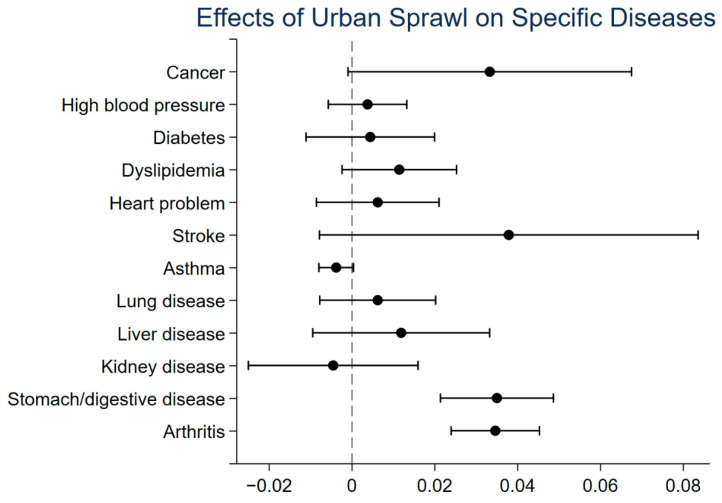
Coefficients and 90% confidence intervals of regressions for each specific disease.

To further examine the effect of urban sprawl on physical health, this study investigates that effect on each specific disease. Figure 1 plots the coefficients and 90% confidence intervals of regressions for each specific disease. The black dots in the figure represent the percentage increase in the probability of getting a specific disease when urban sprawl increases by 1 unit. The horizontal lines in the figure represent the 90% confidence intervals of regression results. In general, the respondents’ risk of getting stomach/digestive disease and arthritis increases significantly. However, urban sprawl does not have a significant effect on other specific diseases. Although urban sprawl does not necessarily increase the risk of each specific disease, it has a significant effect on the overall level of physical health.

**Figure 2:**
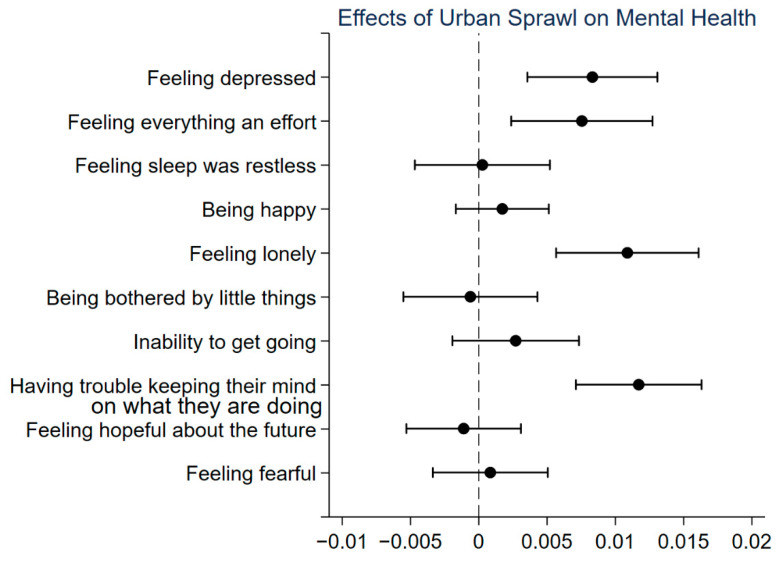
Coefficients and 90% confidence intervals of regressions for each psychological feeling.

To further examine the effect of urban sprawl on mental health, this study examines that effect on each type of psychological feeling reported by respondents in the CESD-10. Figure 2 plots the coefficients and 90% confidence intervals of regressions for each feeling reported by respondents. As urban sprawl increases, the frequency of negative feelings (e.g., the feeling of depressed, feeling that everything is an effort, feeling lonely, and having trouble keeping their mind on what they are doing) increases significantly as the level of urban sprawl rises. However, the effect of urban sprawl on other feelings is not significant. These feelings include the feeling of sleeping being restless, being happy, being bothered by little things, inability to get going, being hopeful about the future, and being fearful. Similar to the previous findings, urban sprawl does not necessarily increase the risk of each negative psychological feeling but has a significant effect on the overall level of mental health.

### 3.3. Heterogeneity Analysis

Urban sprawl may have differential effect on public health for different groups. In this study, we examine the heterogeneity of the health effects of urban sprawl in four dimensions: gender, age, education, and household income level.

#### 3.3.1. Gender Heterogeneity

**Figure 3:**
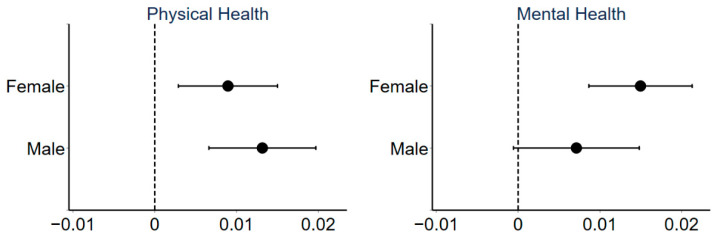
Coefficients and 90% confidence intervals of regressions for different gender group.

Figure 3 shows the regression results of the effect of urban sprawl on public health for different genders. Urban sprawl significantly reduces the physical and mental health of females. Moreover, urban sprawl reduces the physical health of males, but has no significant effect on their mental health.

#### 3.3.2. Age Heterogeneity

**Figure 4:**
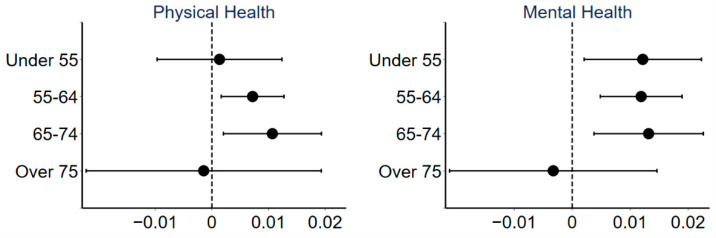
Coefficients and 90% confidence intervals of regressions for different age groups.

Figure 4 shows the regression results of the effect of urban sprawl on public health for groups in different ages. Urban sprawl significantly reduces the physical health of respondents in the 55–74 age group and the mental health of respondents under the age of 75, but has no significant effect on the health of the 75+ age group. This result may be explained by the following reasons. As cities grow, urban spaces mostly expand outward in a circle-like pattern [75]. Most individuals in the older age groups have purchased houses before the massive urban expansion. As cities have expanded, the location of the older group’s housing has become the central urban areas. Meanwhile, older groups have a higher demand for medical facilities, which are mostly concentrated in central urban areas in China. For the relatively younger group, the housing prices in the central urban areas have risen by the time they buy their homes, so they proceed to the spreading suburbs where they can afford the housing prices at lower prices. Thus, in terms of the spatial distribution of the population within the city, the older age groups mostly live in the central city and the relatively younger groups live more in the sprawling suburbs [76]. Therefore, urban sprawl has a significant impact on the health of the relatively younger group and has no significant impact on the 75+ age group.

#### 3.3.3. Education Heterogeneity

**Figure 5:**
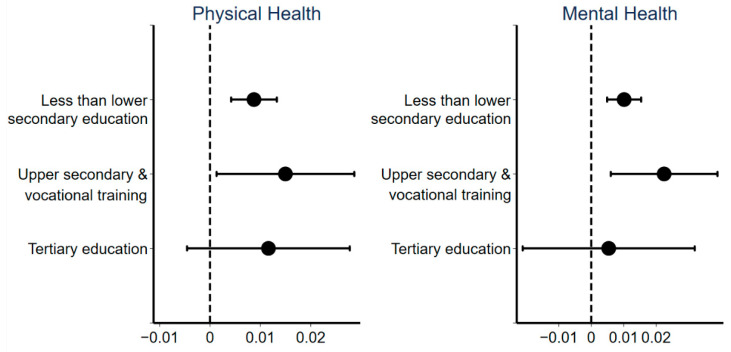
Coefficients and 90% confidence intervals of regressions for different education groups.

Figure 5 shows the regression results of the effect of urban sprawl on public health for groups with different education levels. This study divides the sample into three categories according to the highest education level of the respondents: less than lower secondary education, upper secondary and vocational training, and tertiary education. Urban sprawl has a negative impact on the health of all groups with different education levels. However, this effect is significant in the less educated group but not in the more educated group. Previous research established that the highly educated group in China tends to work in the civil service or public institutions. Most members of that group have local household registration and enjoy housing benefits, and the majority of such housing is in the residential area of the unit which is relatively close to the workplace, thereby facilitating the achievement of work–life balance [77]. By contrast, individuals in the less educated group mostly need to purchase houses on their own in the market, and most of these houses are located in urban sprawl areas. Therefore, urban sprawl does not have a significant impact on the health of the group with high education but has a significant negative impact on the health of the low education group.

#### 3.3.4. Income Heterogeneity

**Figure 6:**
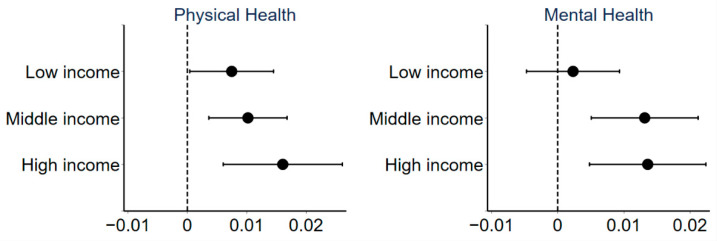
Coefficients and 90% confidence intervals of regressions for different income groups.

Figure 6 shows the regression results of the effect of urban sprawl on public health for groups with different household income levels. This work trisects the sample according to annual household income and classifies respondents into three categories: low income, middle income, and high income. Urban sprawl has a significant negative impact on the physical health of all groups and mental health of the middle- and high-income groups. Moreover, the effect of urban sprawl on respondents’ physical health increases with rising income levels. Most members of the higher income groups are relatively young. As discussed, those individuals generally live in sprawling suburban or new town areas, so urban sprawl has a more significant impact on them. The low-income groups are mostly elderly people who depend on pensions and who mainly live in central or old urban areas where population density is relatively high. Thus, urban sprawl has a significant negative impact on the health of relatively high-income groups but not on the health of relatively low-income.

## 4. Conclusions

This study takes urbanizing China as its research object, uses data from three follow-up surveys conducted by Harmonized CHARLS, and examines the effects of urban sprawl on public health from physical and mental health perspectives. Results show that although urban sprawl does not necessarily increase the risk of each specific type of disease or psychological feeling, it has a significant impact on the overall level of physical and mental health. Further analysis reveals significant heterogeneity in the effects of urban sprawl on the physical and mental health of different groups. Specifically, urban sprawl is detrimental to the physical health of males and females but only has a negative impact on the mental health of females. Among the middle-aged and older groups, the physical and mental health of the younger groups are more vulnerable to damage from urban sprawl. In addition, urban sprawl has a significant negative impact on the health of the low-education group but a very limited impact on the health of the high-education group. From an income perspective, however, the preference for suburban housing among middle- and high-income groups makes their health more vulnerable to the negative effects of urban sprawl than low-income groups living in urban centers.

This study has the following limitations, many of which should motivate future research. First, this study is focused merely on the middle-aged and older groups due to the limited data availability. However, the negative effects of urban sprawl are very likely to be severe for other vulnerable groups such as the poor and children whose health and well-being deserve scholarly and public attentions as well. Second, this study briefly explored several possible mechanisms for urban sprawl to affect public health, such as reduced physical activity [25,36], decreased air quality [15,16], pedestrian-unfriendly built environment. Nevertheless, these mechanisms can hardly be confirmed without substantial empirical tests when data are available in future. Third, since CHARLS only reported the city where the respondent resided but not the exact location within the city, this study can only explore the effect of the overall urban sprawl on residents’ health, but cannot examine the different effects on people living in the city center and those in the suburb. This comparative study is crucial for further study due to the nature of urban sprawl focusing mainly on the spatial pattern of the suburb rather than the city center. Last but not the least, this study is based mainly on survey data and statistical methods with relatively limited theoretical discussion and qualitative analysis. Although data often tell the truth, qualitative or mixed methods are a must for further in-depth investigation on urban spatial structure and residents’ physical and mental health.

## Figures and Tables

**Table 1:** Descriptive statistics of the main variables.

Variable Name	Definition	Obs	Mean	SD	Min	Max
Health						
Physical health	Total number of diseases the respondent had	9232	1.718	1.586	0	10
Mental health	Score of the short form of the Center for Epidemiological Studies Depression scale	9435	7.160	5.757	0	30
Urban sprawl						
Urban sprawl	Urban sprawl index	9803	2.581	3.092	−11.813	19.625
Individual demographic characteristics						
Age		9803	60.502	9.439	45	94
Gender	0 for female; 1 for male	9803	0.471	0.499	0	1
Marriage	0 for separated, divorced, widowed, and never married; 1 for married	9803	0.135	0.342	0	1
Education	0 for less than lower secondary education, 1 for upper secondary and vocational training	9803	0.156	0.363	0	1
Education_2	0 for less than lower secondary education, 1 for tertiary education	9803	0.035	0.183	0	1
Employment	0 for unemployed, retired, or never worked; 1 for currently working	9803	0.536	0.499	0	1
Health behavior variables						
Smoke	0 for never having smoked; 1 for ever smoking	9803	0.404	0.491	0	1
Drink	0 for never having an alcoholic drink in the past; 1 for having had an alcoholic drink in the past	9803	0.428	0.495	0	1
Family structure variables						
Ln(Hhincome)	The sum of all income at the household level	9803	9.713	2.379	0	14.863
Hhcoresd	0 for no child co-resides with respondent; 1 for any child co-resides with respondent	9803	0.549	0.498	0	1
Hhnum	The number of people living in household	9803	3.305	1.576	1	12

**Table 2:** Benchmark regression results.

	Physical Health	Mental Health
	(1)	(2)
Urban sprawl	0.011 ***	0.012 ***
	(4.04)	(3.94)
Age	0.033 ***	−0.000
	(25.13)	(−0.34)
Gender	−0.208 ***	−0.244 ***
	(−6.40)	(−8.26)
Marriage	−0.084 **	0.112 ***
	(−2.53)	(3.74)
Education	0.049	−0.240 ***
	(1.27)	(−7.64)
Education_2	0.048	−0.345 ***
	(0.79)	(−5.56)
Employment	−0.024	−0.045 **
	(−1.49)	(−2.40)
Drink	0.058 ***	−0.005
	(3.75)	(−0.24)
Smoke	0.133 ***	0.061 **
	(5.66)	(2.26)
Ln(Hhincome)	−0.008***	−0.022 ***
	(−3.68)	(−7.18)
Hhcoresd	0.012	−0.006
	(0.83)	(−0.28)
Hhnum	−0.032 ***	0.006
	(−5.42)	(0.93)
Constant	−1.342 ***	2.296 ***
	(−14.22)	(26.62)
N	9232	9435

*** and ** indicate significance at the 1% and 5% levels, respectively.

## Data Availability

The data presented in this study are publicly available as explained in the main text.
